# Supplementary figures and images for: Comparable Genomic Copy Number Aberrations Differ across Astrocytoma Malignancy Grades
Source: Int J Mol Sci. 2019 Mar 12;20(5):1251. doi: 10.3390/ijms20051251 (PMC6429132; doi:10.3390/ijms20051251)

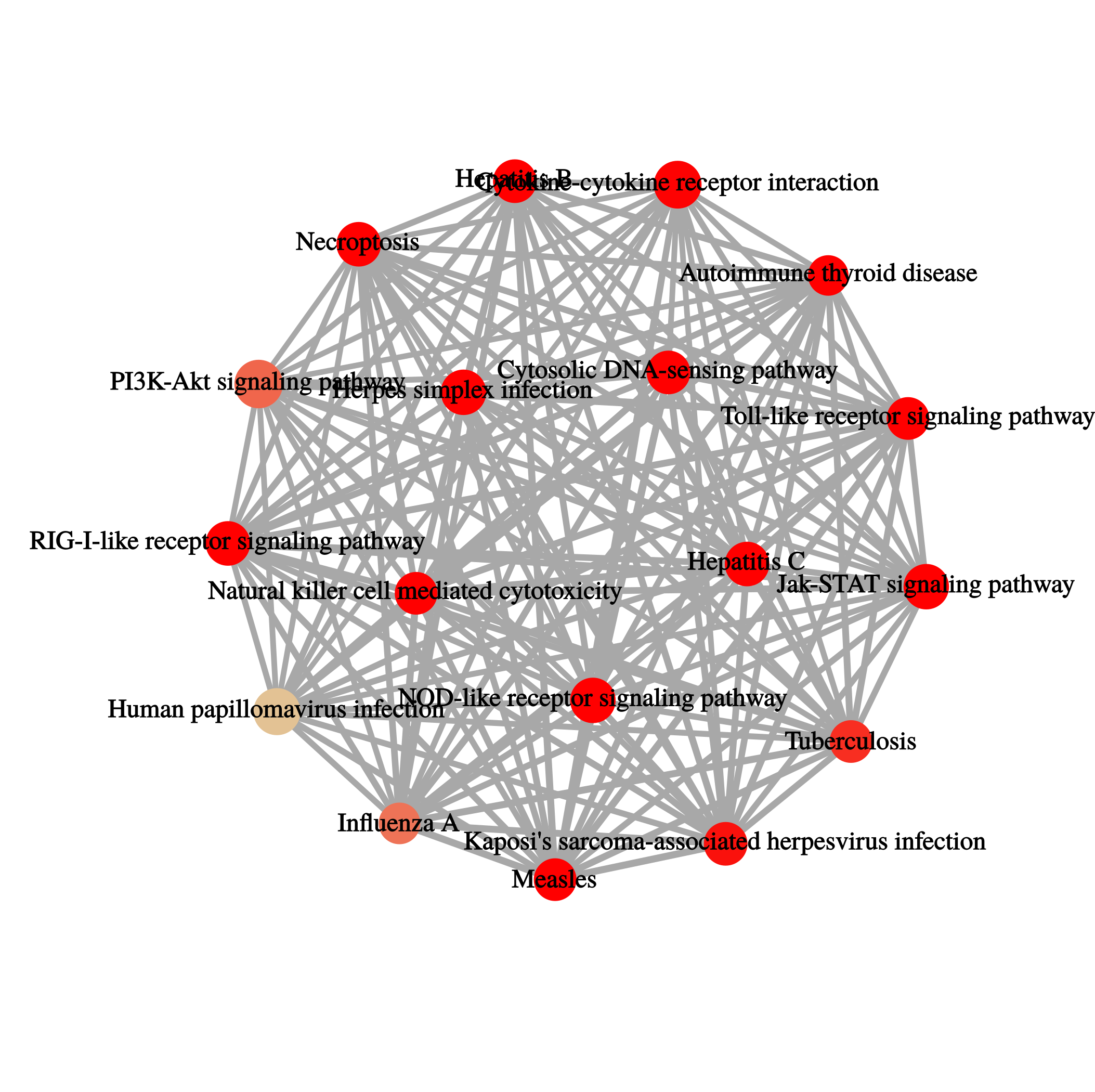

Supplement: Supplementary file 1 [file ijms-20-01251-s001.zip › Supplementary Files/Figure S1.tiff]

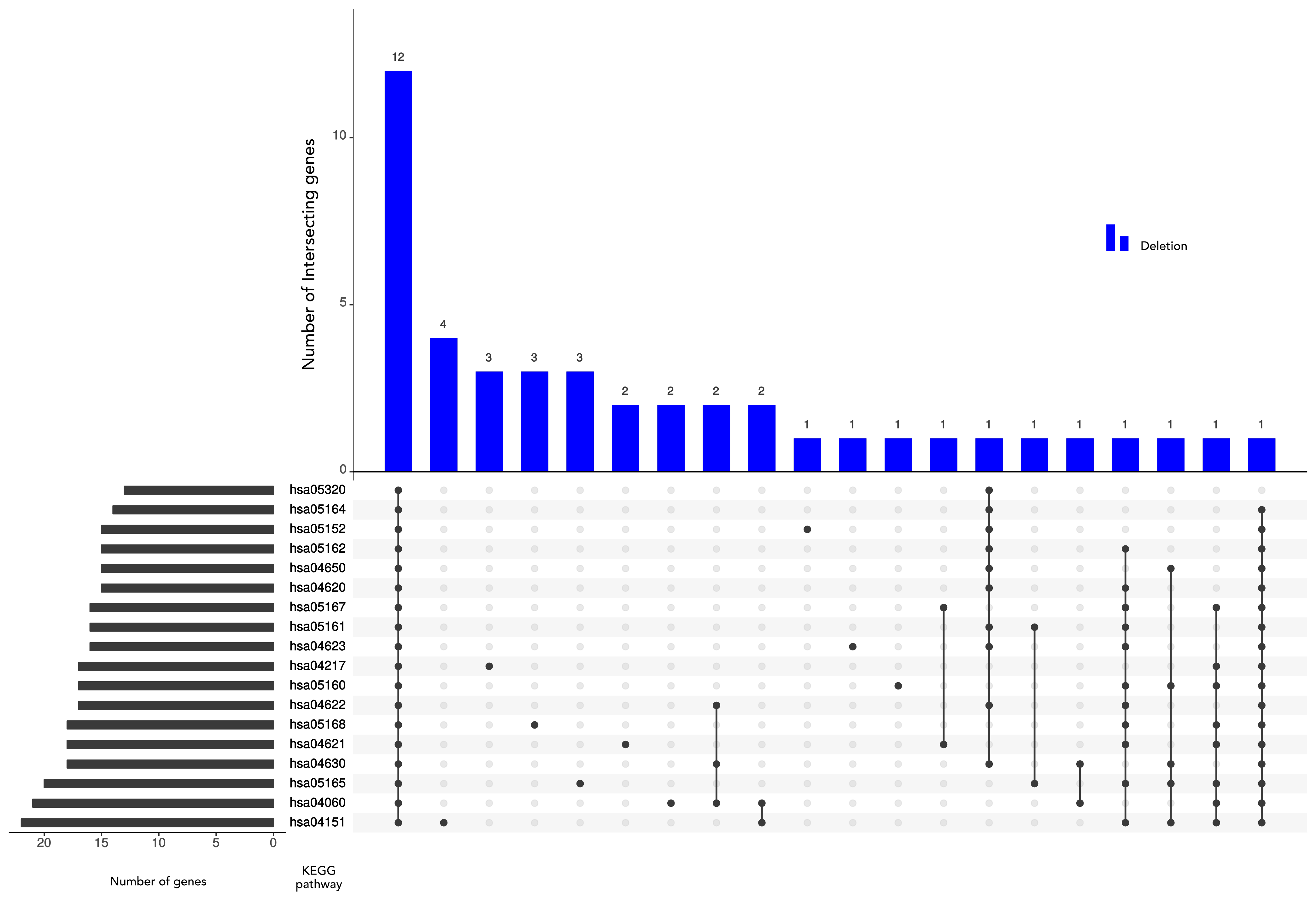

Supplement: Supplementary file 1 [file ijms-20-01251-s001.zip › Supplementary Files/Figure S2.tiff]

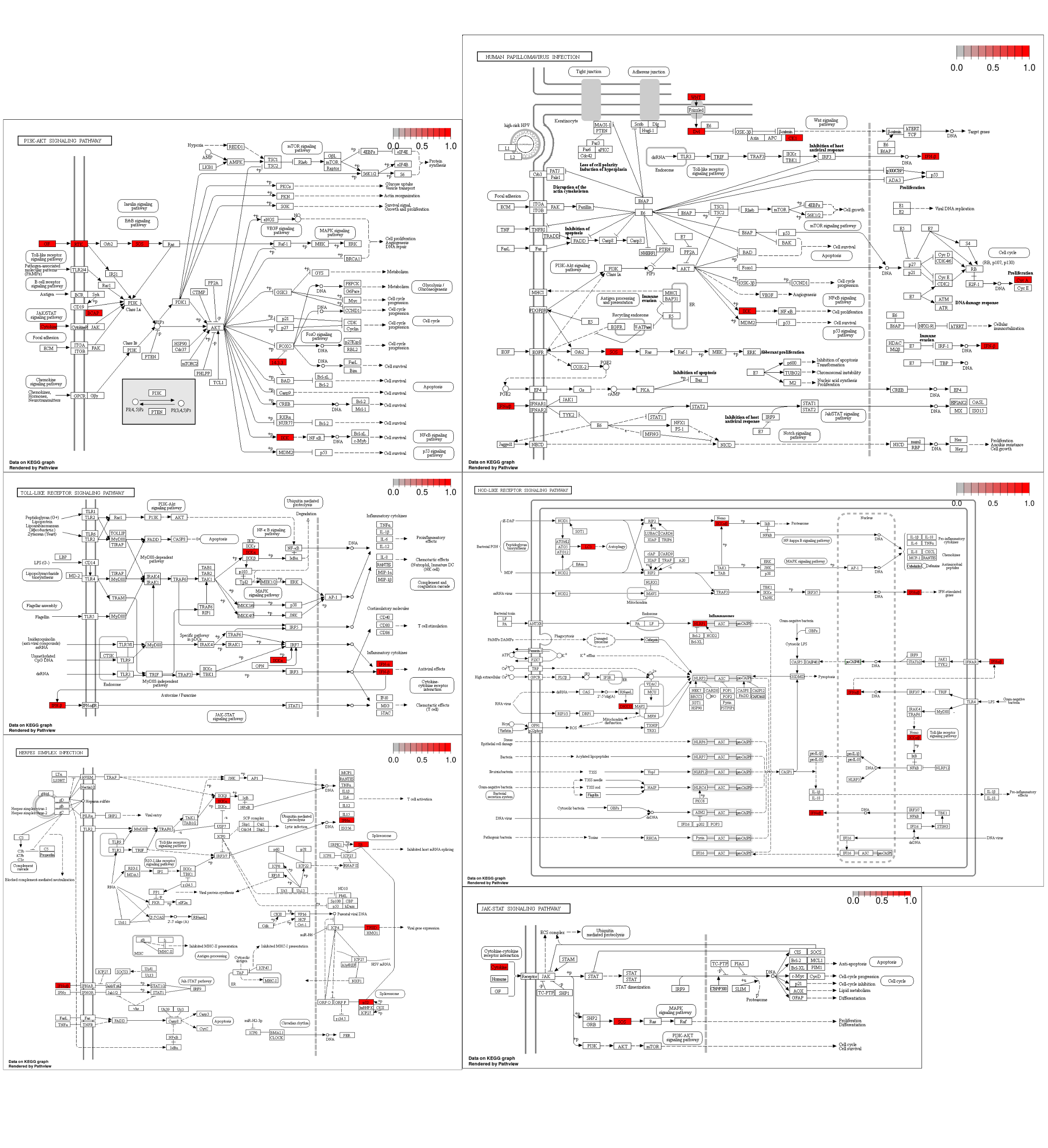

Supplement: Supplementary file 1 [file ijms-20-01251-s001.zip › Supplementary Files/Figure S3.tiff]
